# A system for real‐time monitoring of breath‐hold via assessment of internal anatomy in tangential breast radiotherapy

**DOI:** 10.1002/acm2.13473

**Published:** 2021-11-18

**Authors:** Elena N. Vasina, Peter Greer, David Thwaites, Tomas Kron, Joerg Lehmann

**Affiliations:** ^1^ School of Information and Physical Sciences University of Newcastle Newcastle New South Wales Australia; ^2^ Department of Radiation Oncology Calvary Mater Newcastle Newcastle New South Wales Australia; ^3^ Institute of Medical Physics School of Physics University of Sydney Sydney New South Wales Australia; ^4^ Peter MacCallum Cancer Centre Melbourne Victoria Australia

**Keywords:** central lung distance, deep inspiration breath‐hold (DIBH), EPID, lung depth, monitoring of internal anatomy, real‐time portal imaging, tangential breast radiotherapy

## Abstract

The deep inspiration breath‐hold (DIBH) technique assists in sparing the heart, lungs, and liver during breast radiotherapy (RT). The quality of DIBH is currently assessed via surrogates which correlate to varying degrees with the patient's internal anatomy. Since modern linacs are equipped with an electronic portal imaging device (EPID), images of the irradiated anatomy streamed from EPIDs and analyzed in real time could significantly improve assessment of the quality of DIBH.

A system has been developed to quantify the quality of DIBH during tangential breast RT by analyzing the “beam's eye view” images of the treatment fields. The system measures the lung depth (LD) and the distance from the breast surface to the posterior tangential radiation field edge (skin distance, SD) at three user‐defined locations.

LD and SD measured in real time in EPID images of two RT phantoms showing different geometrical characteristics of their chest wall regions (computed tomography dose index [CTDI] and “END‐TO‐END” stereotactic body radiation therapy [E2E SBRT]) were compared with ground truth displacements provided by a precision motion platform. Performance of the new system was evaluated via static and dynamic (sine wave motion) measurements of LD and SD, covering clinical situations with stable and unstable breath‐hold. The accuracy and precision of the system were calculated as the mean and standard deviation of the differences between the ground truth and measured values.

The accuracy of the static measurements of LD and SD for the CTDI phantom was 0.31 (1.09) mm [mean (standard deviation)] and –0.10 (0.14) mm, respectively. The accuracy of the static measurements for E2E SBRT phantom was 0.01 (0.18) mm and 0.05 (0.08) mm. The accuracy of the dynamic LD and SD measurements for the CTDI phantom was –0.50 (1.18) mm and 0.01 (0.12) mm, respectively. The accuracy of the dynamic measurements for E2E SBRT phantom was –0.03 (0.19) mm and 0.01 (0.11) mm.

## 1 INTRODUCTION

When applied during breast cancer radiotherapy (RT), the deep inspiration breath‐hold (DIBH) technique helps to spare the organs at risk such as the heart, the lungs, and, in right‐breast irradiation, the liver.^1^, ^2^ Deep inspiration expands the lung and displaces the upper abdominal organs away from the chest wall. The quality of DIBH is currently assessed by various techniques, for example, by tracking an external marker block placed on the patient's chest or abdomen,^3^ by monitoring the patient's skin surface with optical surface monitoring systems,^4^ by controlling the breathing,^5^, ^6^ or by employing the treatment room lasers and tattoos on the patient's abdomen.^7^ A low‐cost in‐house system for monitoring DIBH with an industrial laser distance meter was reported by Jensen et al.^8^ All such systems have in common is that they are not assessing the clinically relevant part of the patient's anatomy but a more or less motion correlated surrogate. Additionally, the precision of DIBH monitoring with these systems depends on selection of the region of interest on the patient's skin or choice of the location for the reflective marker block on the patient's abdomen and these choices might be suboptimal due to the patient's size and shape and obstruction by the gantry of the linac.

**FIGURE 1:**
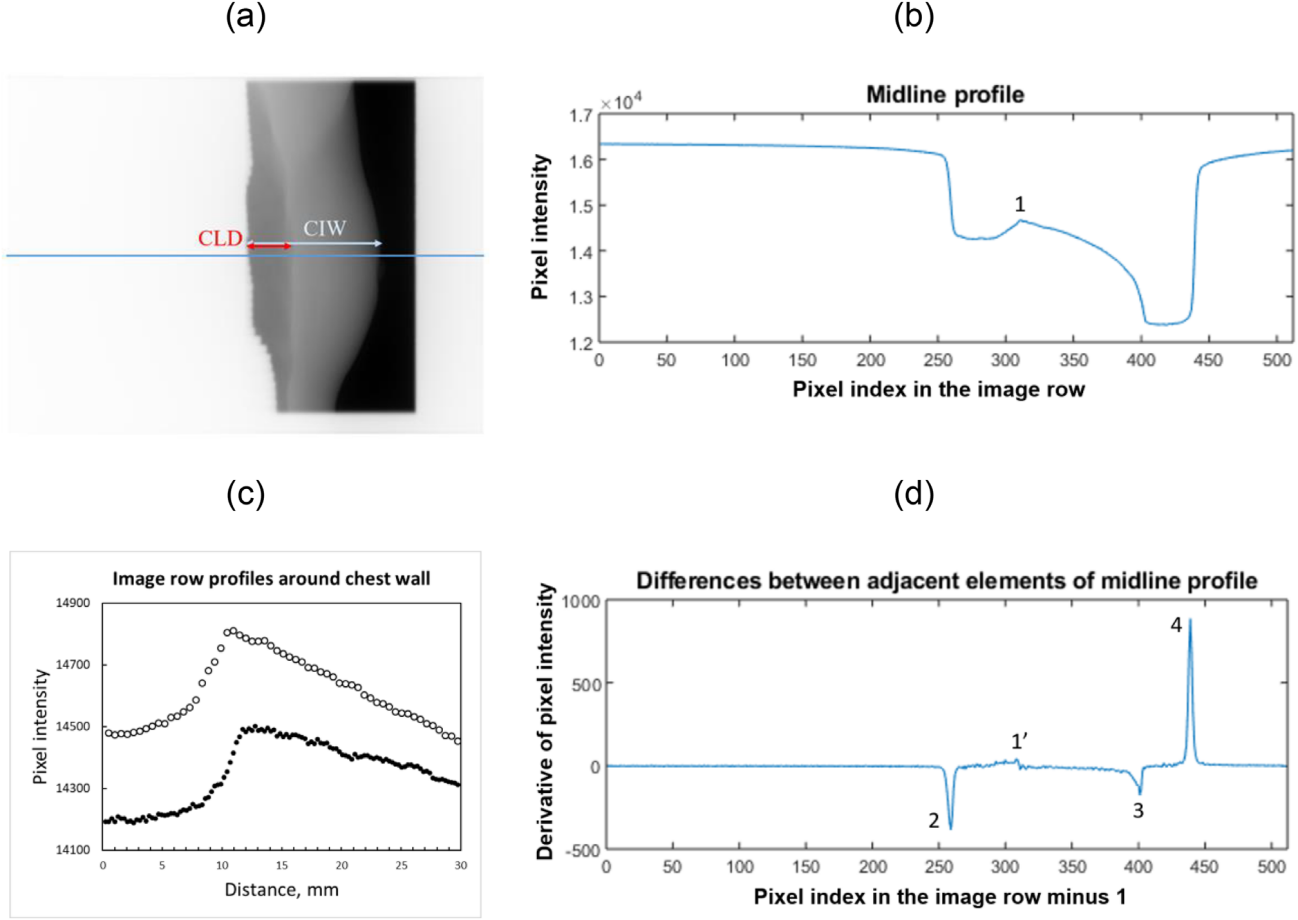
(a) MV portal image of a tangential breast field: the red segment indicates the central lung distance (CLD) also called the central lung depth (LD); the light blue segment shows the central irradiated width (CIW) which is called central skin distance (SD) in this study; the blue line shows the position of the image row used in the description of the image processing algorithm. (b) The profile of the image row shown by the blue line shown in (a), the pixel intensity versus the pixel index: peak 1 shows the location of the chest wall. (c) Typical chest wall peaks seen in MV images of tangential breast fields: a sharp peak (open symbols) and a broad peak (solid symbols). (d) The first derivative of the profile shown in (b). The peak denoted by 1′ is the peak of the chest wall, the negative peak denoted by 2 is the peak of the posterior field edge, peak 3 shows the location of the breast surface, and peak 4 is the peak of the anterior field edge. X‐size of the pixels is 0.52 mm

All modern linear accelerators are equipped with an electronic portal imaging device (EPID). Using images provided by the EPID to retrospectively assess the position of the irradiated internal anatomy of the patient during treatment has been reported on. Geometrical parameters such as the central lung distance (CLD) and the central irradiated width (CIW) measured in EPID images of tangential breast fields (see Figure 1a) were employed to retrospectively study positioning errors and inter‐ and intra‐fraction motion,^9^, ^10^ to estimate via CLD the dose to the ipsilateral lung,^11^ and to find correlation between CLD and ipsilateral lung dose parameters.^12^ Retrospective measurements of CLD in Megavolt (MV) EPID images of patients acquired continuously during tangential breast RT with DIBH levels monitored by the Varian's RPM system found significant deviations from the intended treatment for some patients.^13^, ^14^

A number of in‐house image processing tools were reported for retrospective assessment of the quality of DIBH, displaying the trajectories of free breathing motion, and assessment of heart radiation exposure in MV images of tangential breast fields. Doebrich et al.^14^ utilized an interactive image analysis routine (MATLAB, MathWorks, Natick, MA, USA) to calculate the CLD as the distance between the inflection point of the pixel intensity profile of the posterior field edge and the adjacent local maximum representing the bony chest wall. Jensen et al.^15^ developed a program to detect the position of the chest wall edge applying a Canny filter. Poulsen et al.^16^ designed an algorithm to automatically segment the heart shadow in images of breast tangents. Software, with the algorithm based on the PeakFinder function of MATLAB, for measuring the CLD was presented in Lehmann et al.^17^ Intra‐fractional motion of the breast surface and the bony chest wall at the central beam axis were measured retrospectively in cine EPID images of tangential breast fields for free breathing RT by Hong et at.^18^ using software based on a pattern matching algorithm.

Changes of CLD, CIW and lung depth (LD) and irradiated width (IW), which is called here skin distance (SD), at other locations in the treatment field can be measured in real time. In this work, LD is the distance from the posterior radiation field edge to the “middle” of the bony chest wall, and SD is the distance from the posterior radiation field edge to the skin surface of the breast. These parameters can help to evaluate the quality of DIBH via the internal anatomy. Movements of internal anatomy close to the target conceptually have a stronger correlation to the target than correlation between movements of external surrogates and the target. Besides, as the EPID‐based approach utilizes the treatment beam to create the images, no additional radiation imaging dose is given to the patient.

This study evaluates the accuracy and precision of a new system for real‐time measurements of the LD and the distance from the skin to the posterior field edge (SD) in MV portal images of tangential breast treatments. The new system monitors the breath‐hold when the radiation treatment beam is on and the images of the treatment field are being acquired, thus an additional system (e.g., based on the room lasers) is needed to guide the patient's breath‐hold prior to beam‐on. The system consists of new software for measuring LD and SD at multiple user‐selected locations and a frame grabber application for real‐time acquisition of MV EPID frames. The software for evaluating LD and SD was written in C#; its image processing algorithm can handle traditional open tangential fields as well as more complex fields with multileaf collimator (MLC) leaves blocking the breast surface. This work covers the system design and the validation of its accuracy. Clinical tests of the new system, which are ongoing and are being conducted in parallel with the real‐time position management (RPM) and Catalyst+ breath‐hold monitoring systems, will be briefly mentioned but are beyond the scope of this work.

## 2 METHODS

### 2.1 Portal MV images of breast treatments and images of RT phantoms

MV EPID images of tangential breast fields acquired in DICOM format in the course of DIBH treatments locally and in RT departments in New Zealand and Australia (TROG 14.04 HART trial, https://www.trog.com.au/1404‐HART) were used for the development of the image processing algorithm. In addition, MV images of RT phantoms recorded during real‐time tests produced a supplementary set of images.

The TROG 14.04 trial investigated the implementation of DIBH in a clinical setting. As part of the trial, MV images of tangential breast fields (500+ images of 62 study patients) had been collected. The images were obtained with a range of EPIDs to verify the accuracy of patient positioning before or during treatment. They included medial–lateral (ML) and lateral–medial (LM) projections of left and right breast treatments. Shadows of the heart or liver were present in some of the images. There were several post‐mastectomy images.

### 2.2 System components

The system comprises two components: (i) an in‐house application (named C‐DOG) for real‐time acquisition of single MV EPID frames in TIFF format and (ii) software for reading the TIFF files and measuring the LD and SD at three user‐defined locations during tangential breast RT. The latter software is named LEILA (Live EPID‐based Inspiration Level Assessment).

#### 2.2.1 Software and image processing algorithm for measuring the LD and SD in real time during tangential breast RT

To enable robust and fast real‐time measurements in a clinical environment, the algorithm of the LEILA software was coded in C# (Microsoft® Visual Studio 2017). The MV images of tangential breast fields and RT phantoms (Section 2.1) were used to test the code.

LEILA's prototype was developed in MATLAB, where it was employed for retrospective analysis of the tangential breast fields. It contained a method to distinguish between ML and LM views as the gantry angle was not available for all images.

For the real‐time version of LEILA, the gantry angle is provided by the C‐DOG application. Additional parameters needed for measurements of LD and SD are the collimator angle, which is available from the DICOM plan file, and the pixel size of the EPID at the isocenter. The latter is calculated from the pixel spacing of the EPID and the source to image distance (SID), the default SID is 150 cm.

LEILA reads the image of the most recent TIFF file saved in real time in a user‐specified folder and analyses image row profiles and their first derivatives (the differences between the adjacent elements) at multiple user‐specified locations. Figure 1a,b shows a sample MV EPID image of a tangential breast field and the profile of its midline row. The peak denoted by 1 is the peak of the chest wall profile. Figure 1c shows two typical examples of chest wall profiles seen in images of tangential breast fields: a sharp peak (open symbols) and a broad peak (solid symbols). The pixel index of peak 1 provides the location of the bony chest wall for the calculations of the LD in LEILA. Figure 1d shows the first derivative of the row profile shown in Figure 1b: the peak denoted by 1′ is the peak of the bony chest wall profile, the negative peak denoted by 2 is the peak of the posterior field edge, peak 3 is related to the breast surface, and peak 4 is the peak of the anterior field edge.

To find the upper and lower borders of the radiation field (patient superior–inferior direction), the algorithm employs a threshold technique. The expression for the threshold coefficient, *b*, is given by

(1) $$b=I_{\max}-\left\lfloor \frac{I_{\max}-I_{\min}}{d}\right\rfloor .$$

The floor function is defined as ⌊*x*⌋ = max{*n* ∈ *Z*: *n* ≤ *x*}, where *Z* denotes integer numbers. The *I*_max_ and *I*_min_ are the maximum and minimum pixel intensities in the image. The threshold denominator, *d*, needs to be determined experimentally for a particular EPID using retrospective images of breast treatments or phantoms. For the portal imagers of the TROG trial, the values of *d* were found to be in the interval [2.5; 6], with the most frequent value being 2.5. For the locally acquired EPID images in DICOM format, *d* = 2.5 confirmed the lengths of the radiation fields with submillimeter accuracy (the collimator angle was at 0^o^). To obtain the binary image matrix, the pixel intensities above *b* are replaced with value 1 and the remaining pixel intensities are replaced with value 0. The upper field border is found as the first row of the binary image having the mean row intensity below 0.99, the lower border of the radiation field is found in a similar way.

The vertical size of the radiation field is computed for the binary image and is compared with the planned value, which is available via the DICOM plan file. If the radiation field partially extends beyond the top or bottom boundary of the EPID, the algorithm will calculate the location of the field midline taking into account the part of the field extending beyond the boundary. Only up to 25% of the vertical field size are allowed to extend beyond the border of the EPID, this allows for measurements of LD and SD along the three default lines. The EPID coordinates should be chosen (if possible) such that the projected radiation field does not extend beyond the boundaries of the EPID.

**FIGURE 2:**
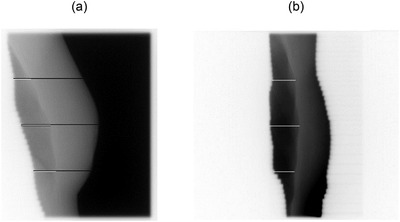
Portal MV images of tangential breast fields. (a) The distances from the skin to the posterior field edge (skin distances, SDs) are shown by the long black lines; the lung depths (LDs) are shown by the white lines. The inferior LD and inferior SD are crossing the heart shadow. (b) The superior, central, and inferior LDs detected for the partially blocked anatomy: the MLC leaves are blocking the skin

Assessments of LD and SD are done at three user‐defined lines of interest identified by their distance from the midline (central line of the radiation beam). The default positions of the three lines are: (i) the midline plus, (ii) the superior, and (iii) inferior lines calculated as half‐way from the midline to the upper and lower borders, respectively. Figure 2a shows a portal image with LDs and SDs drawn at the three default locations (superior, midline (central), and inferior), and Figure 2b shows LDs measured on an image with the breast surface blocked by the MLC leaves. The drawing of LDs and SDs was implemented in LEILA's prototype as part of the analysis for visual verification of the algorithm's performance.

For each line of interest the algorithm assesses the presence of breast surface in the image. It uses the average of three adjacent rows of pixels (averaged profiles) at the position of the line. Using three rows was found to be a suitable compromise between reducing noise and maintaining peak information. A third‐order one‐dimensional median filter is used to filter noise in the averaged profiles, and a 1 × 3 and 1 × 5 average filter is used to filter the first derivatives of the averaged profiles.

If the breast surface is present, the program calculates SD as the distance between the posterior edge of the field (peak 2, Figure 1d) and the breast surface (peak 3, Figure 1d), otherwise SD will be reported as 0 mm. For the same average of three adjacent rows of pixels, LD is calculated as the distance from the pixel index of the maximum pixel intensity of the bony chest wall region (peak 1, Figure 1b) to the pixel index of the posterior edge of the radiation field (peak 2, Figure 1d). Greater variability of measured LD is expected for broad peaks of the bony chest wall, a typical example of a broad peak is shown in Figure 1c. The algorithm assumes that the bony chest wall peak is the brightest part of the image row next to the posterior border of the radiation field (Figure 2a,b).

Image rotation by a known angle can be applied if, for example, the collimator angle is not at 0^o^. To rotate an image by an angle specified in radians, the following transformation of the pixel coordinates^19^ is employed:

(2) $$\begin{array}{ccc} x^{\prime } & = & \left(x-\frac{w}{2}\right)\cos\left(\alpha \right)-\left(y-\frac{h}{2}\right)\sin\left(\alpha \right)+\frac{w}{2},\\ y^{\prime } & = & \left(x-\frac{w}{2}\right)\sin\left(\alpha \right)+\left(y-\frac{h}{2}\right)\cos\left(\alpha \right)+\frac{h}{2}, \end{array}$$

where *x*’ and *y*’ are the new pixel coordinates, *x* and *y* are the initial pixel coordinates, *α* is the angle of rotation, *w* is the width of the image in pixels, and *h* is the height of the image in pixels.

#### 2.2.2 Software for real‐time acquisition of MV images

In‐house image acquisition software C‐DOG was used to acquire TIFF files of single MV frames in real time.^20^ The software is using a frame grabber card Matrox Solios SOL 2M EV CLB (Matrox Electronics, Dorval, Quebec, Canada) connected to the clinical system control computer port to receive EPID frames. Apart from the pixel data of a single MV frame, additional information is recorded in the TIFF files but it was not used in this study. The image acquisition software records 10–13 images per second depending on the linac: it takes approximately 76 ms to save a TIFF file on a Varian TrueBeam StX linac and 105 ms on a Varian Clinac (Varian Medical Systems, Palo Alto, CA, USA).

### 2.3 Data acquisition

Figure 3a shows the overall workflow of the system including the real‐time recording of MV frames (C‐DOG software) and real‐time measurements of LD and SD (LEILA software). When image acquisition starts, the most recent EPID frame (TIFF file) is analyzed by the system each round. The system measures and displays in real time three LD and three SD parameters. The rate of image processing is four to five images per second if no image rotation is performed (collimator angle 0^o^), and two to three images per second with image rotation. The latency of the system is <350 ms: it is estimated as the sum of the time delays of C‐DOG (100 ms) and of LEILA (<250 ms) applications. The time delay of LEILA includes the test that the most recent TIFF file is ready to be read.

FIGURE 3(a) Workflow of real‐time recording of MV frames with C‐DOG software and real‐time measurements of lung depth (LD) and skin distance (SD) with Live EPID‐based Inspiration Level Assessment (LEILA) software. (b) Photo of the experimental set‐up with the CTDI phantom: the Varian Clinac with the gantry at 270^o^, the electronic portal imaging device (EPID), and the CTDI phantom placed on the motion platform. The precision motion platform was used to simulate changes in LD and SD during unstable breath‐hold. (c) MV portal image of the CTDI phantom. (d) MV portal image of the E2E SBRT phantom. The LD (red line) and the SD (cyan line) are shown schematically in (c) and (d). On MV images of the E2E SBRT phantom, LD and SD were measured along image rows showing the ribs. (e) Chest wall peaks in MV images of the CTDI (green dots) and E2E SBRT (open squares) phantoms and a broad peak of a patient (black dots). The graphs were shifted vertically and horizontally for ease of comparison

**Figure:**
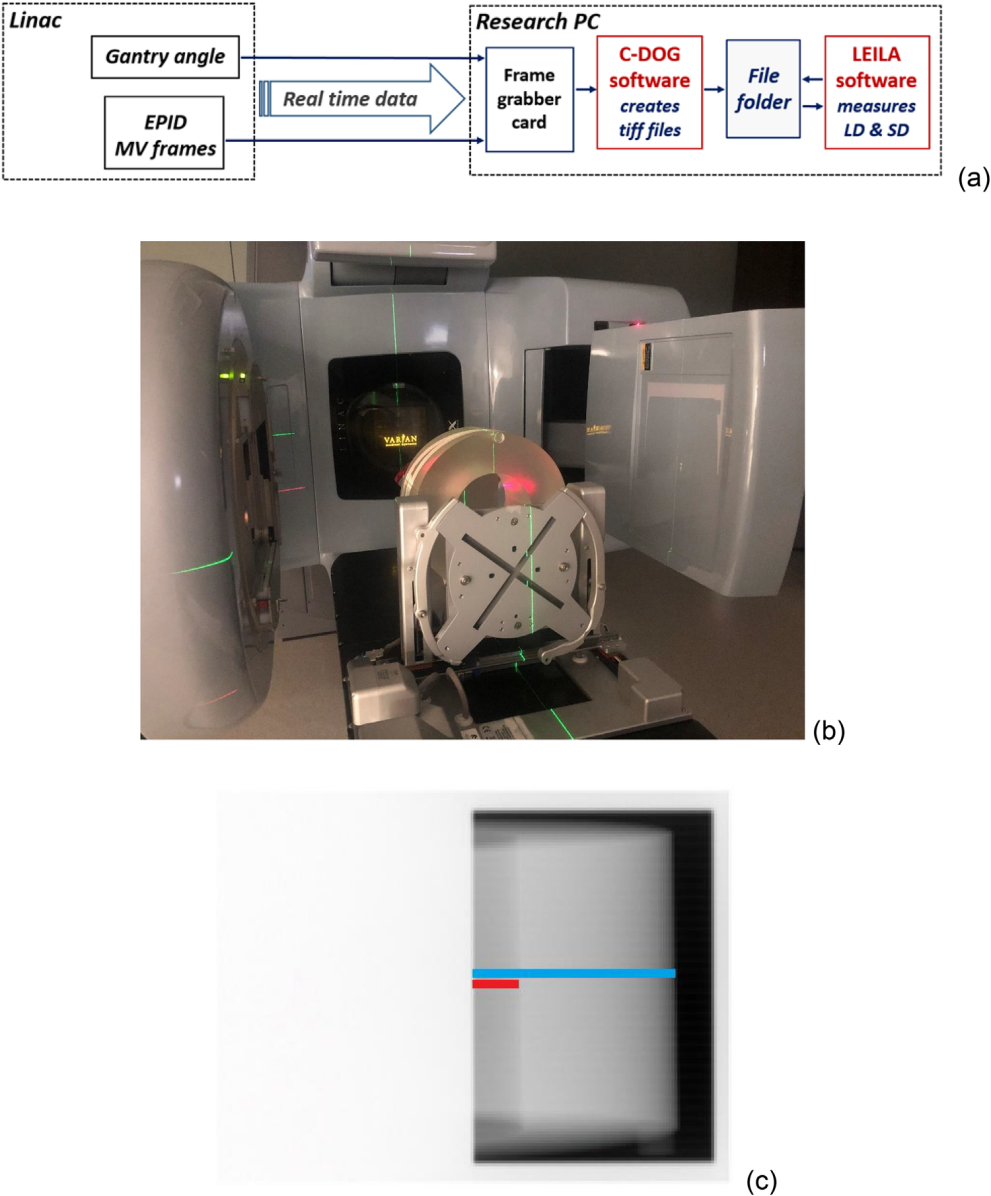

**Figure:**
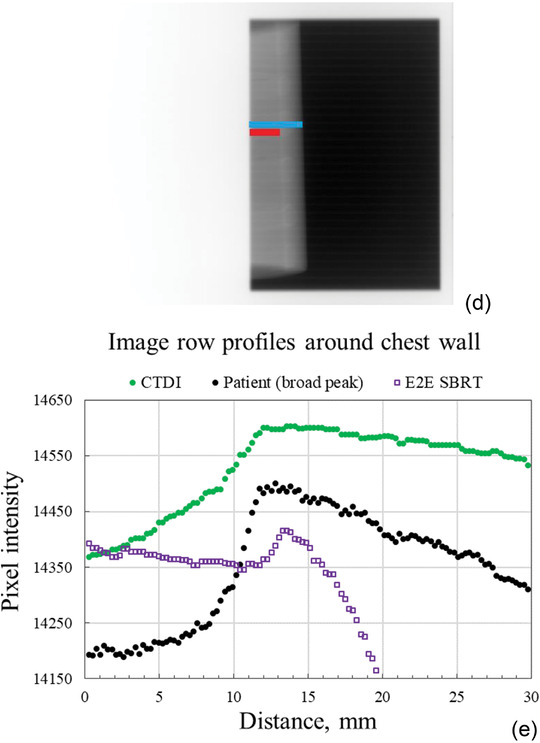

#### 2.3.1 Experimental set‐up

Real‐time measurements of LD and SD were carried out on a Varian Clinac. MV images of RT phantoms used in the development and testing of the algorithm included images acquired on a Varian TrueBeam StX. The linacs are equipped with an amorphous silicon EPID: an aS1000 (1024 × 768 pixel array, 0.391 mm pixel pitch) and an aS1200 (1190 × 1190 pixel array, 0.336 mm pixel pitch), respectively.

The system assessment tests were performed with the annulus component of a PMMA computed tomography dose index (CTDI) phantom (ring‐shaped phantom, IBA Dosimetry GmbH, Schwarzenbruck, Germany), and with an anthropomorphic thorax body “END‐TO‐END” stereotactic body radiation therapy (E2E SBRT) phantom (CIRS, Norfolk, VA, USA). The thickness of the wall of the CTDI phantom is approximately 8 cm. The CTDI phantom displays a broad peak of the chest wall region, although it is twice as broad as the broad peak of patients (Figure 3e), while the E2E SBRT phantom presents a sharp peak of its chest wall region, similar to that of patients (Figure S1a).

To displace the phantoms in a precise way during the dynamic and static tests, a programmable motion platform with positioning accuracy better than 0.5 mm (Hexamotion, ScandiDos, Uppsala, Sweden) was employed.

#### 2.3.2 Radiation delivery characteristics

A rectangular field (180 × 125 mm^2^ defined by the jaws) of 6 or 18 MV X‐rays was delivered to the phantoms using the Varian Clinac with the gantry at 270^o^ and the collimator at 0^o^. The time of irradiation was about 300 s to accommodate longer experiments than typical DIBH treatment times. The dose rate was 600 Monitor Units (MU) per minute.

The gantry angle of 270^o^ was chosen to simplify data analysis and to make the plane of the EPID coplanar with the vertical motion of the RT phantoms measured in parallel with a commercial surface‐monitoring system.

#### 2.3.3 Motion trajectories and the ground truth

For experiments with the CTDI phantom, it was placed on its side on the motion platform and fixed with adhesive tape (Figure 3b). The center of the vertical axis of symmetry of the phantom coincided with the treatment isocenter. For experiments with the E2E SBRT phantom (Figure S1c), it was positioned on the motion platform at an angle to simulate the incident beam angle typically employed during the tangential breast RT. During irradiations, the phantoms were displaced along the *Z*‐axis, orthogonally to the surface of the treatment couch. Figure 3c,d shows the LD and the SD drawn schematically on the portal images of the CTDI and E2E SBRT phantoms. On images of the E2E SBRT phantom, LD and SD were measured along the image rows showing the ribs. Image rows without ribs do not have the row profile characteristics similar to those observed in the images of breast tangents.

Two motion trajectories were applied to the phantoms: (i) upward and downward steps with static poses between each step and the programmed amplitude of steps being 3 mm, and (ii) sine wave motion with a programmed peak‐to‐peak amplitude of 6 mm. To ascertain that the motion platform produced the correct motion characteristics, measurements with the surface monitoring system Catalyst+ HD (C‐RAD, Uppsala, Sweden) were carried out.

**FIGURE 4:**
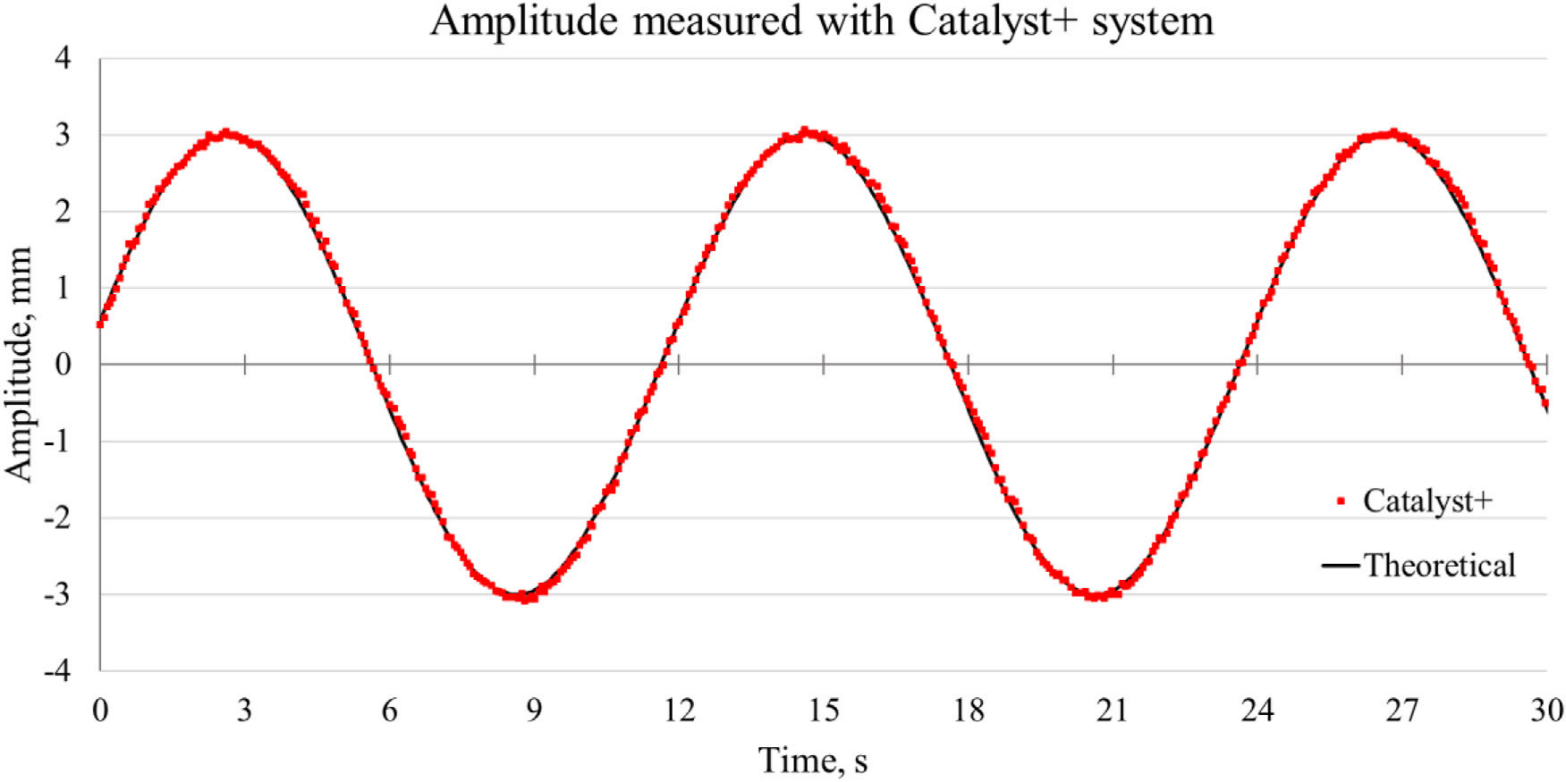
Quality assurance of the sine wave motion of the motion platform with Catalyst+ system. The motion amplitude of the CDTI phantom placed on top of the motion platform as measured by Catalyst+ (red dots) and the theoretical (programmed) sine function fitted to the experimental data (solid line). The theoretical sine function had a peak‐to‐peak amplitude of 6 mm and a time period of 12 s

During sine wave motion with the peak‐to‐peak amplitude of 6 mm and the time period of 12 s, relative vertical coordinates of the top of the phantom's side facing upwards were measured by the Catalyst+ system. The variation of measured amplitudes with time followed a sine curve and was shifted downward to make the midline of the sine curve coincide with the time axis. The sine wave function, which the motion platform was programmed to move, was fitted to the measured curve (Figure 4) using Microsoft Excel's Data Analysis tool. Subsequently, the programmed motion was assumed to be accurate and it served as the ground truth (expected function) for the LD and SD measurements.

**FIGURE 5:**
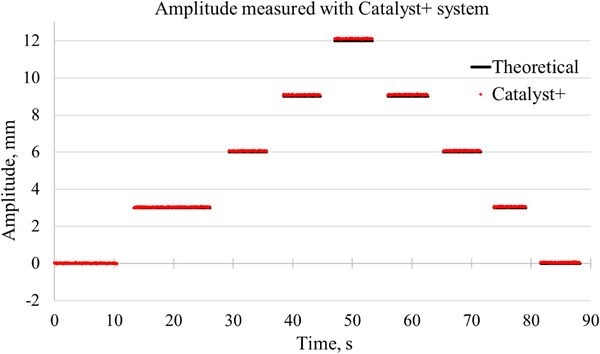
Quality assurance of the motion platform with Catalyst+ system: the amplitudes of the steps of the motion platform measured during the static poses. Data obtained with Catalyst+ system (red dots) and the theoretical (programmed) amplitudes (solid lines). The programmed amplitude of the steps was 3 mm

Similarly, the amplitudes of the steps of the motion platform were measured by Catalyst+ system during the static poses (Figure 5). A very small drift upward is present in the Catalyst+ data when the amplitude increases; further tests showed that it was due to hysteresis in the motion of the precision motion platform and thermal drift of the Catalyst+ system (which was within the specifications).

### 2.4 Real‐time measurements of LD and SD with RT phantoms

As described above, the system measures the LD and the distance from the skin to the posterior field edge by analyzing the intensity profiles and the first derivatives of user‐selected image rows. To quantify the accuracy and precision of the system, three LD parameters (superior, central, inferior) and three corresponding SD parameters were measured during the sine wave motion of the phantoms as well as during the static poses between vertical displacements of the platform. Since the tolerances for the surface‐based techniques evaluating the quality of DIBH in breast cancer RT are typically set at 3–5 mm, the amplitude of the sine wave motion and the magnitude of the vertical displacements of the motion platform in this study were set at 3 mm.

### 2.5 Quantifying the accuracy and precision of LD and SD measurements

The accuracy and precision of LD and SD measurements were evaluated as the mean and standard deviation (s.d.) of the differences between the measured values and the corresponding ground truths. Root‐mean‐square (RMS) errors were also calculated.

## 3 RESULTS

### 3.1 Quality assurance of the motion platform

The RMS error between the measured curve by Catalyst+ and the theoretical sine function presented in Figure 4 was found to be 0.07 mm. The RMS error calculated between the programmed amplitudes of the steps and those measured with the Catalyst+ system (Figure 5) was found to be 0.06 mm. The theoretical amplitudes were taken as the ground truth in the subsequent analysis of the accuracy and precision of measured LD and SD.

### 3.2 LD and SD measured during static poses

**FIGURE 6:**
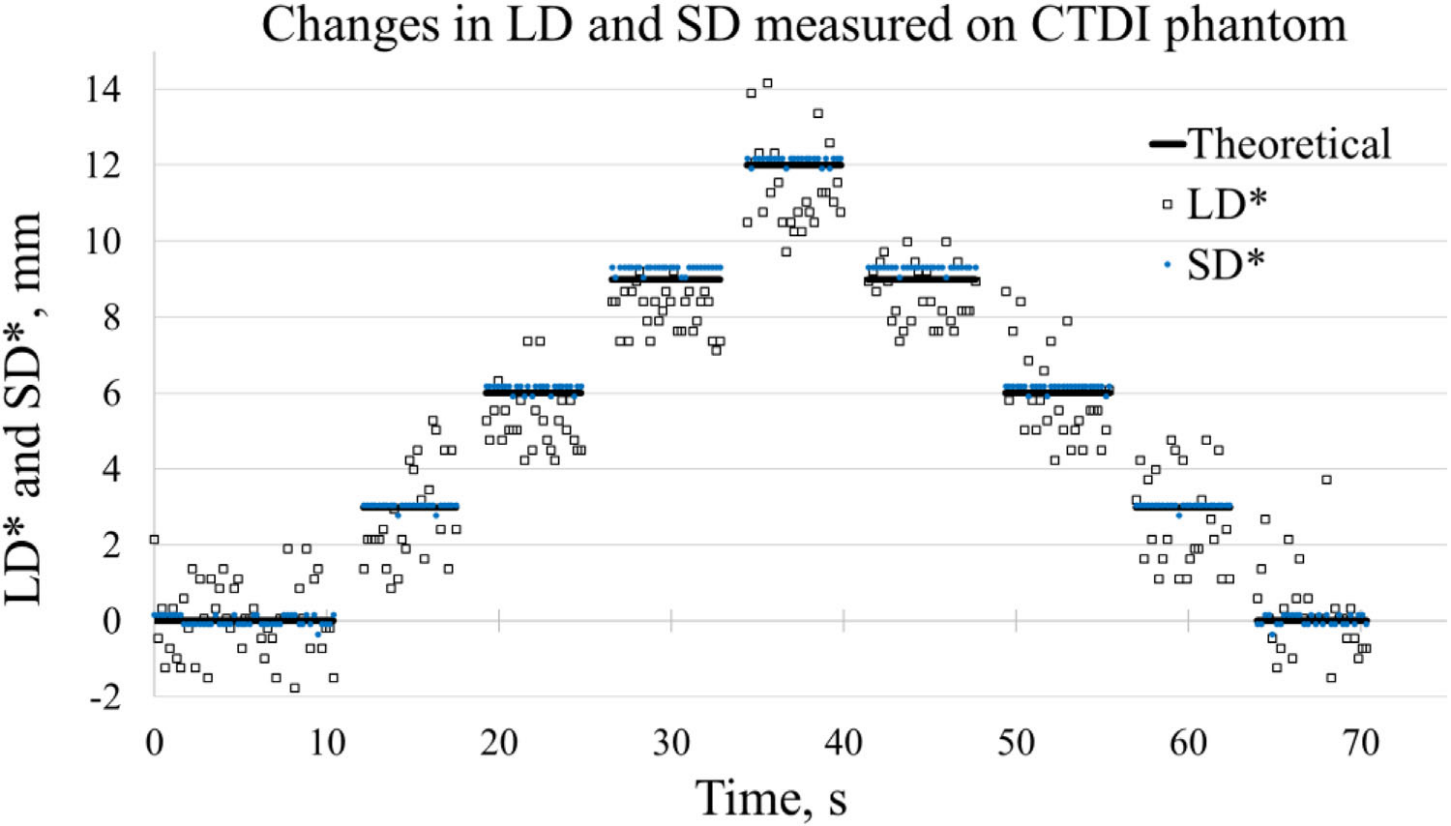
Differences between measured lung depth (LD) and skin distance (SD) values and their start positions versus time on the CTDI phantom during the static poses (LD*, SD*). The start positions were calculated for the time interval from 0 to 10 s. LD* (open squares); SD* (blue dots). The expected values are shown by the black lines. The programmed amplitude of each step of the motion platform was 3 mm

**FIGURE 7:**
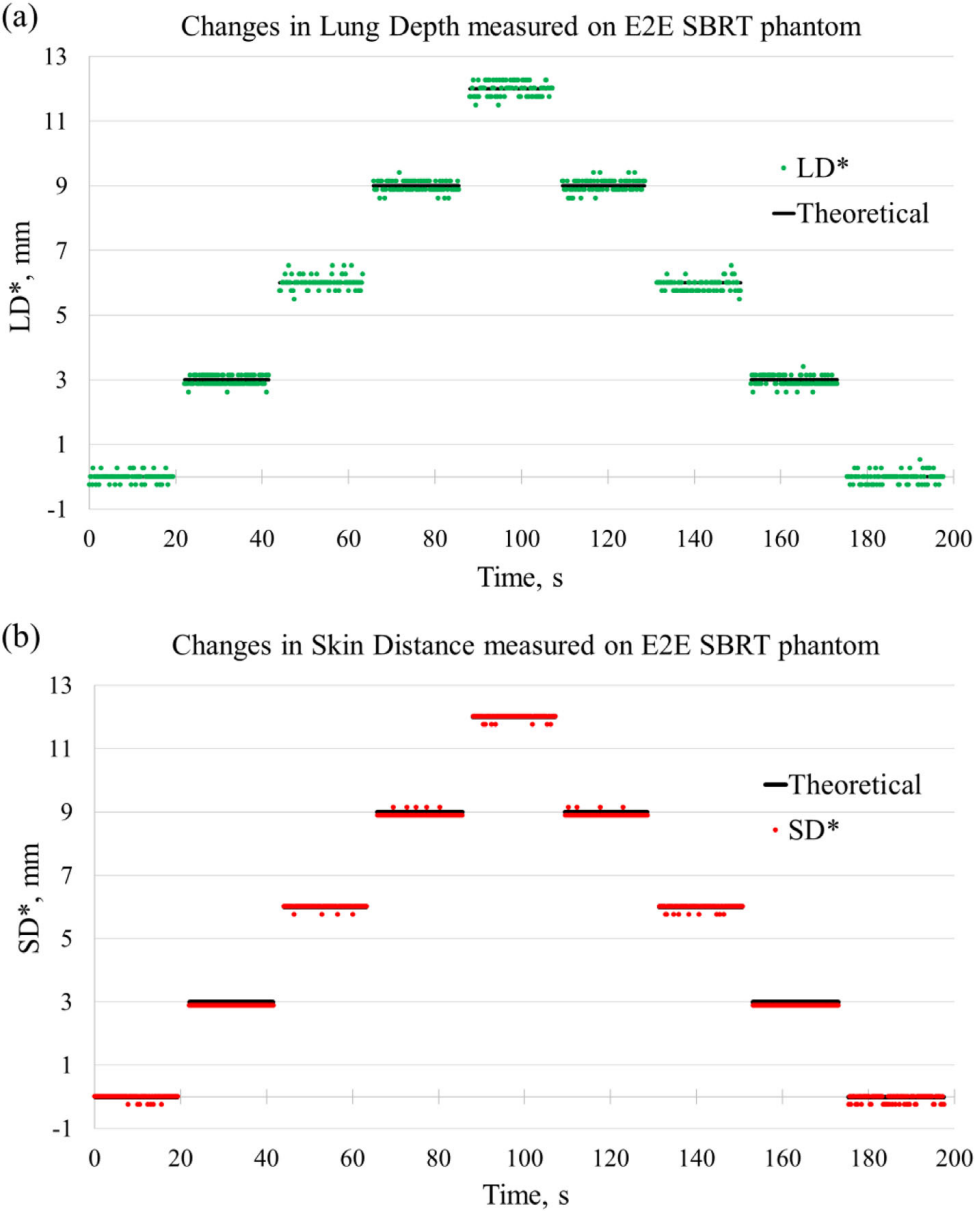
Differences between measured lung depth (LD) and skin distance (SD) values and their start positions on the E2E SBRT phantom during the static poses (LD* and SD*). The start positions were calculated for the time interval from 0 to 20 s. (a) LD* (green dots); (b) SD* (red dots). The theoretical (programmed) values are shown by the black lines

The measured values of LD and SD are presented as differences from their respective start positions and are denoted by LD* and SD*. Figure 6 compares LD* and SD* measured on the CTDI phantom with the applied shifts. Figure 7 presents LD* and SD* measured on the E2E SBRT phantom.

### 3.3 LD and SD measured during sine wave motion

**FIGURE 8:**
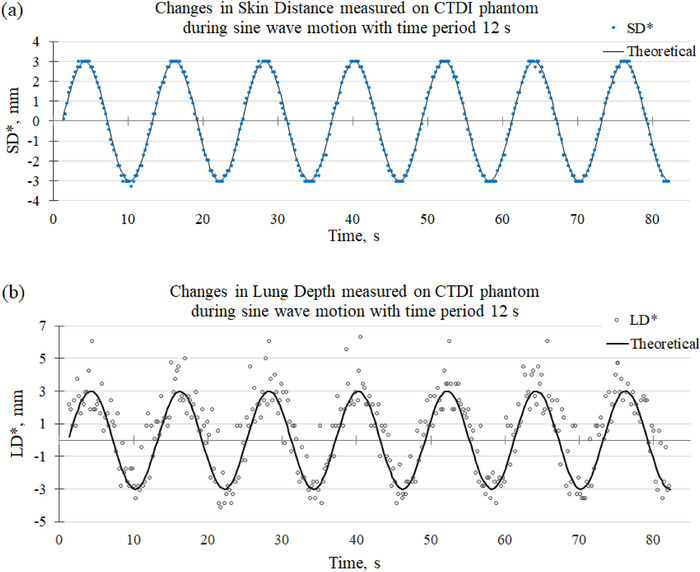
(a) Changes in skin distance (SD) (SD*, blue dots) measured in images of the CTDI phantom during the sine wave motion. The solid line represents the theoretical (programmed) sine function (peak‐to‐peak amplitude 6 mm, time period 12 s) fitted to the experimental data. (b) Changes in lung depth (LD) (LD*, open circles) measured in the images of the CTDI phantom during the sine wave motion. The solid line represents the theoretical (programmed) sine function. The phase shift of the theoretical sine function was taken equal to that of the sine function fitted to the SD* data shown in (a)

Figure 8a shows changes in SD values measured during sine wave motion of the CTDI phantom (denoted by SD*). The data points were shifted downward to make the midline of the sine curve coincide with the time axis and the theoretical sine function was fitted to them. The peaks of the experimental SD* appear clipped (blue circles) because the pixel size of the EPID being 0.261 mm (at the isocenter) is too large to clearly display the programmed sine function.

A similar data analysis was carried out for LD values measured concurrently with SD during the sine wave motion of the CTDI phantom. The measured LDs were shifted downward to make the midline of the sine curve coincide with the time axis, and the difference was denoted by LD*. Figure 8b shows the LD* and the theoretical (programmed) sine curve. The overestimated amplitude is due to the broad peak of the CTDI phantom (Figure 3e, green dots).

**FIGURE 9:**
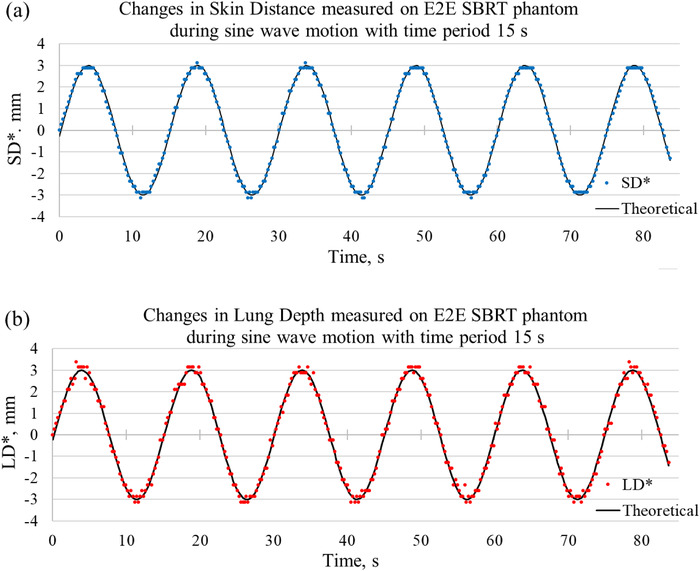
(a) Changes in skin distance (SD) (SD*, blue dots) measured in images of the E2E SBRT phantom during the sine wave motion (peak‐to‐peak amplitude 6 mm, time period 15 s). (b) Changes in lung depth (LD) (LD*, red circles) measured during the sine wave motion. LD and SD were measured along image rows showing the ribs (Figure 3d)

Figure 9a shows changes in SD values measured during sine wave motion of the E2E SBRT phantom (denoted by SD*) and the theoretical sine function fitted to the data points. The theoretical (programmed) sine wave motion had a peak‐to‐peak amplitude of 6 mm and a time period of 15 s. Figure 9b shows the changes in LD (LD*) measured concurrently with SD. In addition, measurements of changes in SD and LD values during sine wave motion with a time period of 5 s were made (Figure S2).

### 3.4 Accuracy and precision of LD and SD measurements

The mean values and s.d. of differences between the ground truths and the experimental data points as well as the corresponding RMS errors for measurements of the LD and the SD on the CTDI and the E2E SBRT phantoms are summarized in Tables 1 and 2, respectively.

**TABLE 1:** Accuracy and precision of measurements of lung depth (LD) and skin distance (SD) in portal MV images of the CTDI phantom

	SD		LD	
Applied motion	Mean (SD) difference (mm)	RMS error (mm)	Mean (SD) difference (mm)	RMS error (mm)
Static poses between vertical steps (amplitude 3 mm)	–0.10 (0.14)	0.18	0.31 (1.09)	1.13
Sine wave (peak‐to‐peak amplitude 6 mm, period 12 s)	0.01 (0.12)	0.12	–0.50 (1.18)	1.28

Abbreviation: RMS, root‐mean‐square.

**TABLE 2:** Accuracy and precision of measurements of lung depth (LD) and skin distance (SD) in portal MV images of the E2E SBRT phantom

	SD		LD	
Applied motion	Mean (SD) difference (mm)	RMS error (mm)	Mean (SD) difference (mm)	RMS error (mm)
Static poses between vertical steps (amplitude 3 mm)	0.05 (0.08)	0.10	0.01 (0.18)	0.18
Sine wave (peak‐to‐peak amplitude 6 mm, period 15 s)	0.01 (0.11)	0.11	–0.03 (0.19)	0.19
Sine wave (peak‐to‐peak amplitude 6 mm, period 5 s)	0.01 (0.15)	0.15	0.08 (0.24)	0.25

Abbreviation: RMS, root‐mean‐square.

### 3.5 Real‐time measurements of the central lung depth in patients

**FIGURE 10:**
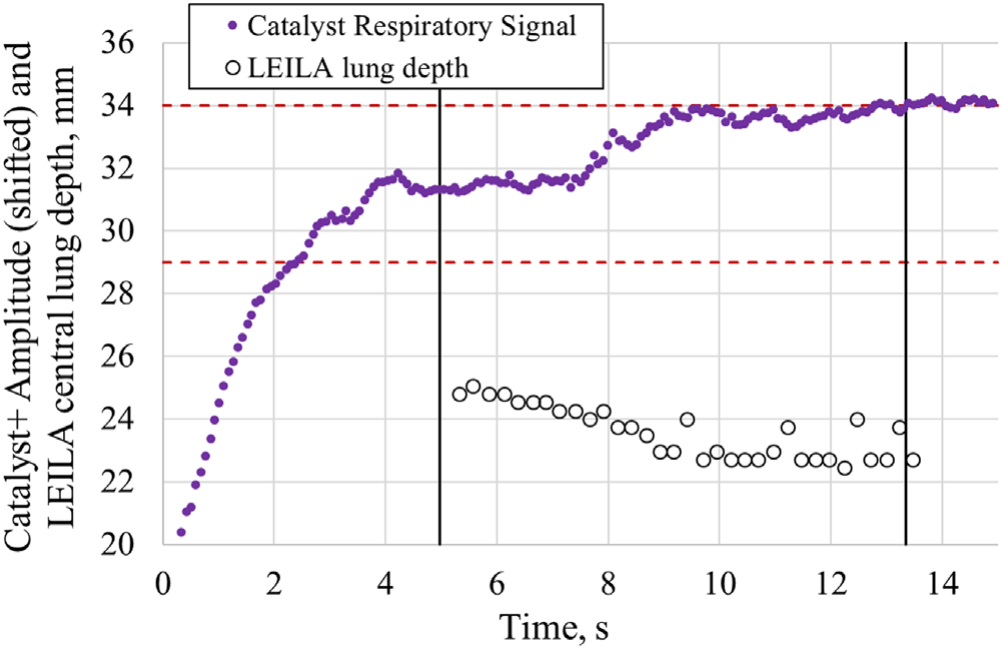
The time traces of inspiration and deep inspiration breath‐hold (DIBH) signals recorded with Catalyst+ system and Live EPID‐based Inspiration Level Assessment (LEILA) software (only during the beam‐on time interval). The solid circles show the time interval from 0 to 5 s when the patient takes in a deep breath (recorded with Catalyst+). The open circles show the central lung depth (CLD) measured in electronic portal imaging device (EPID) images of the patient with LEILA software. The two vertical lines show the beam‐on time interval. Initial values of the Catalyst+ amplitudes were shifted for convenience of comparison with CLD data

An observational study with the new system was conducted in parallel with Catalyst+ system during tangential breast RT with DIBH. A variation of the central lung depth (CLD) measured during one beam is presented in Figure 10 for a left‐sided breast cancer patient, two more examples are shown in Figure S3. The planning CLD value was measured on the patient's bony digitally reconstructed radiograph (DRR); the 5 mm tolerance window for CLD for the treatment beam was from 17 to 22 mm.

Prior to the treatment, image‐guided set‐up verification with a single EPID image taken in DIBH was carried out and couch shifts were applied, therefore, the superior–inferior set‐up error is expected to be minimal. Rotational set‐up error could not affect the direction of the time evolution of CLD. Figure 10 shows that the time traces of CLD and the amplitude of a Catalyst+ monitored spot on the patient's skin moved in the opposite directions. This demonstrates that vertical motion of the spot on the patient's skin measured with the Catalyst+ system does not always have a positive correlation with the real motion of the bony chest wall.

In addition, CLD parameters were measured for three patients with left breast treatments and their DIBH guided with the Catalyst+ system. The patients’ positions were corrected before each treatment fraction based on single EPID images (MV port films). Average CLDs per beam were calculated and compared to their planning values. For 20 beams of 62 studied, the average CLDs were found to be outside the tolerance window, and the maximum deviation was 2.9 mm (that is 5.4 mm from the expected CLD).

## 4 DISCUSSION

The standard EPID verification protocol when a single EPID image is taken in DIBH prior to the treatment helps in set‐up verification but it can suffer from systematic errors. During treatment, the breath‐hold is usually monitored with surrogates or the patient's skin surface with the location of the spot for DIBH monitoring often outside of the patient's chest. This could lead to significant errors.^13^, ^14^

Real‐time assessment of the LD in MV EPID images can conceptually offer a stronger correlation to the motion of the target for monitoring of the quality of DIBH during tangential breast RT than the external surrogate‐based techniques. This study introduces a new system for real‐time measurements of LD and SD and evaluates its accuracy and precision. The latency of the system is comparable with that of a real‐time tumor monitoring system.^21^

As summarized in Tables 1 and 2, the measurements of SD are more accurate and precise than that of LD. This is due to the higher contrast between the phantom and air: the peak of the phantom surface is sharp and well defined, similar to peak 3 in Figure 1d. A lesser contrast between the peak of the chest wall and the surrounding tissues makes the measurements of the LD slightly less accurate. The CTDI phantom produces a broad peak of the chest wall profile (the peak is twice broader that a typical broad peak of the patient), thus further affecting the accuracy of the LD measurements. In contrast, the E2E SBRT phantom produces a sharp peak of the chest wall profile and that is reflected in the submillimeter accuracy and precision of the measurements of LD for this phantom.

**FIGURE 11:**
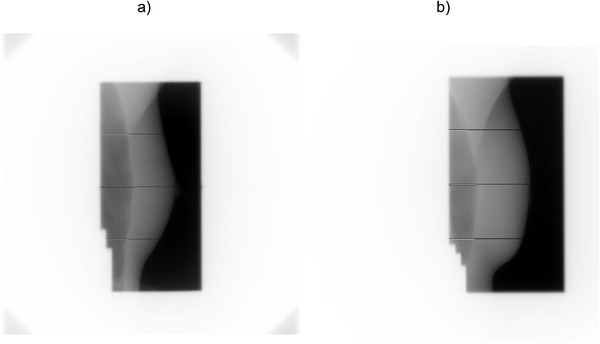
Examples of the performance of the image processing algorithm. (a) The example of how the algorithm measures the central skin distance (also called the central irradiated width): the measured parameter does not include the nipple. (b) The tangential breast field showing the high pixel intensity of the chest muscle in the upper quarter of the image which can interfere with the measurements of the lung distance (shown by the upper while line)

Image rows for measurements of LD and SD need to be selected carefully. The nipple (Figure 11a) and the change in pixel intensity produced by the chest muscle (the superior SD is crossing such a region in the upper quarter of Figure 11b) might interfere with the measurements.

Tracking the SD can expand the LD‐based monitoring of DIBH to the cases with little or no lung in the EPID images. A retrospective analysis of DIBH tangential breast fields showed that SD can be a reliable breath‐hold indicator (Section S4 and Table S1) but it can vary due to breast swelling or the skin surface can be blocked by the MLC leaves.

First clinical real‐time measurements of the CLD with the new system taken in parallel with monitoring of the patient's DIBH with Catalyst+ system demonstrated that motion of the patient's skin measured with the Catalyst+ did not always have a positive correlation with the real motion of the chest wall at the central plane of the radiation beam.

## 5 CONCLUSION

Accuracy and precision of the new system for real‐time measurements of the LD and the distance from the skin to posterior field edge (SD, also called IW) in MV images of tangential breast fields were evaluated experimentally with two RT phantoms displaying different geometrical characteristics of the chest wall region. The SD was shown to be a more precise measure than the LD, although the SD can vary due to breast swelling during RT.

The new system is geometrically accurate and fast for real‐time tracking of the LD and SD at three user‐specified locations. It is able to analyze two to five images per second depending on collimator rotation.

The system is expected to improve the quality of DIBH treatments (heart dose sparing) while not negatively impacting the workload for treatment planning and delivery by RT staff. It holds the potential for accurate treatment delivery without additional imaging dose and using existing imaging hardware.

## AUTHOR CONTRIBUTIONS

*Creation of the new software, software testing, performing measurements/experiments design of the experiments, writing of the manuscript*: Dr. Elena N Vasina. *Participation in the concept of the project, participation in the design of experiments, critical revision of the manuscript*: Prof. Peter Greer. *Participation in the concept of the project, critical revision of the manuscript*: Prof. David Thwaites. *Acquisition of the clinical images (Australia and New Zealand), approval of the version of the manuscript*: A/Prof. Tomas Kron. *Design of the project and funding acquisition, performing measurements/experiments design of the experiments, critical revision of the manuscript*: A/Prof. Joerg Lehmann.

## CONFLICT OF INTEREST

The authors declare no conflict of interest.

## ETHICAL STATEMENT

The use of patient images in this work has been reviewed and approved by the Hunter New England Human Research Ethics Committee, Australia (2020/ETH00720).

## Supporting information

SUPPORTING INFORMATION
