# Supplementary material for: A system for real‐time monitoring of breath‐hold via assessment of internal anatomy in tangential breast radiotherapy
Source: J Appl Clin Med Phys. 2021 Nov 18;23(1):e13473. doi: 10.1002/acm2.13473 (PMC8803293; doi:10.1002/acm2.13473)
Supplement: Supplementary file 1 — SUPPORTING INFORMATION [file ACM2-23-e13473-s001.docx]

Supplemental material for

**A system for real time monitoring of breath hold via assessment of internal anatomy in tangential breast radiotherapy**


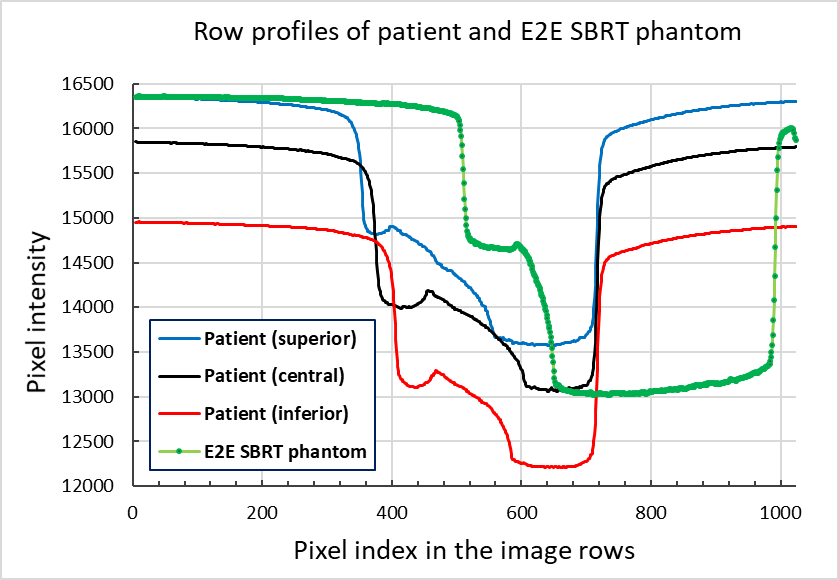
 (a)


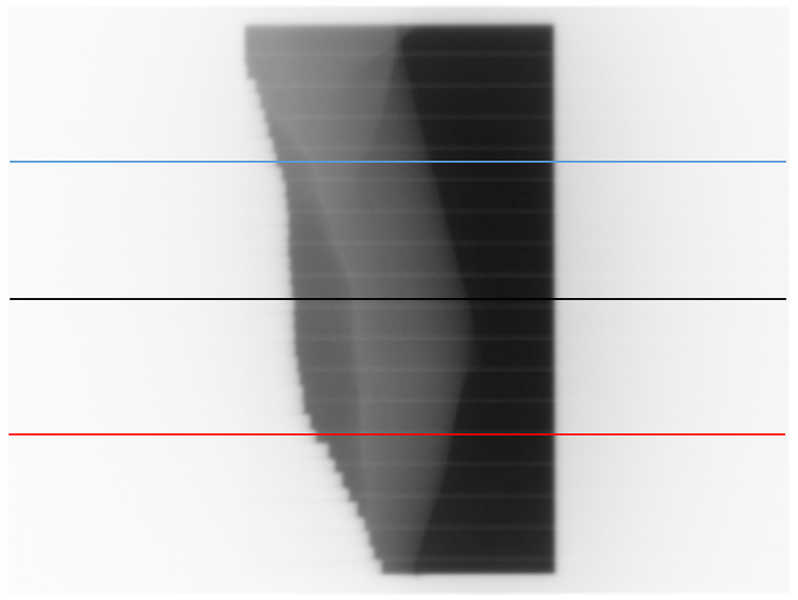
 (b)


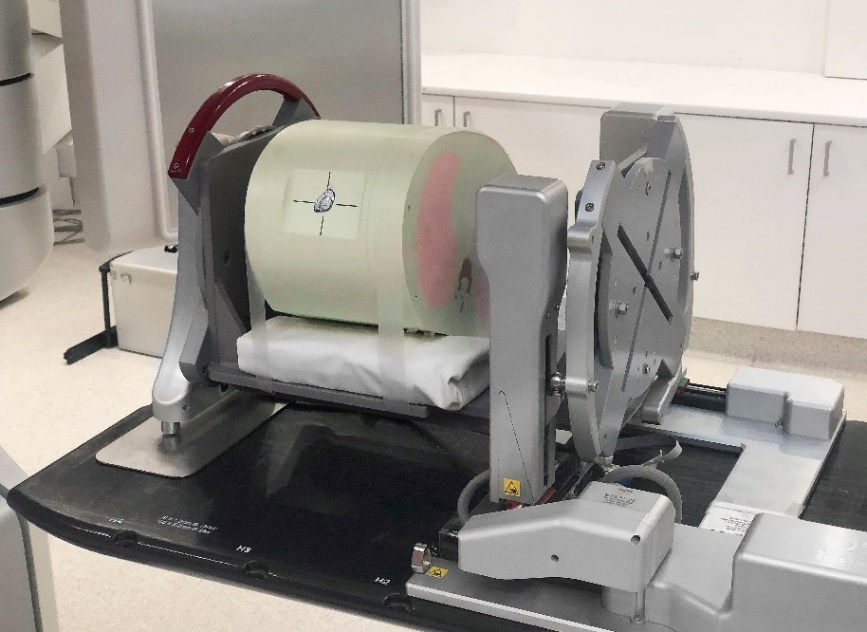
 (c)

Fig. S1. (a) Examples of image row profiles of a patient and the E2E SBRT phantom. The graphs of the central and inferior row profiles of the patient were shifted downwards in pixel value for ease of comparison. (b) Portal MV image of the tangential breast field of the patient for which the row profiles (at the default locations: superior, central, and inferior) are presented in (a). (c) Photo of the experimental setup for the E2E SBRT phantom.


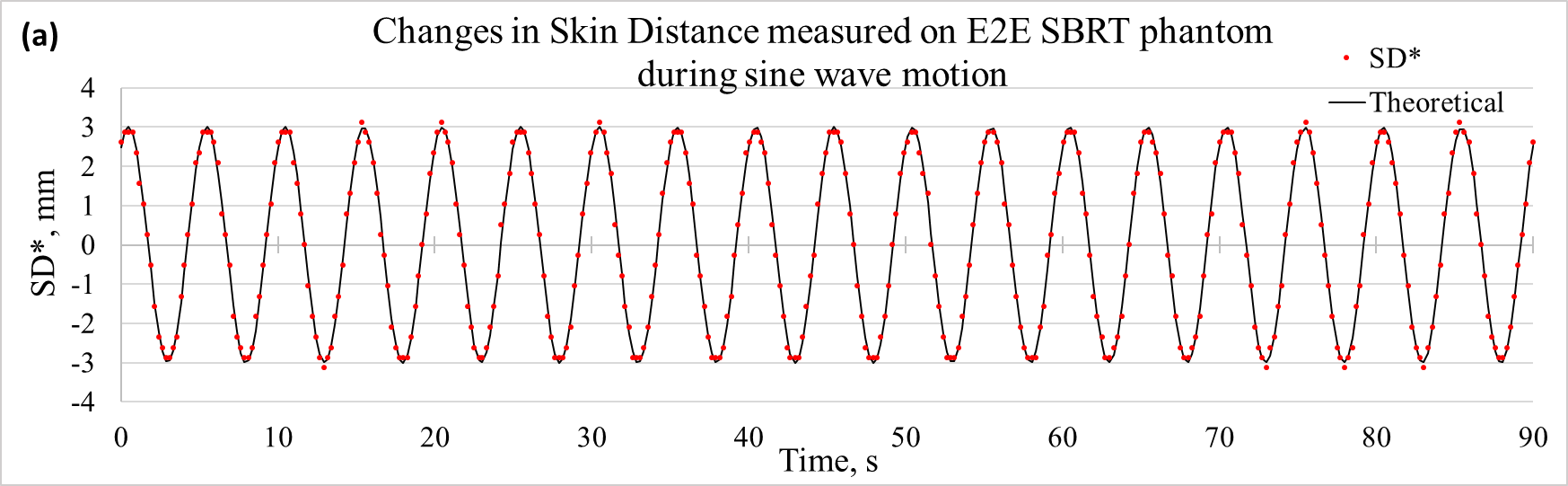


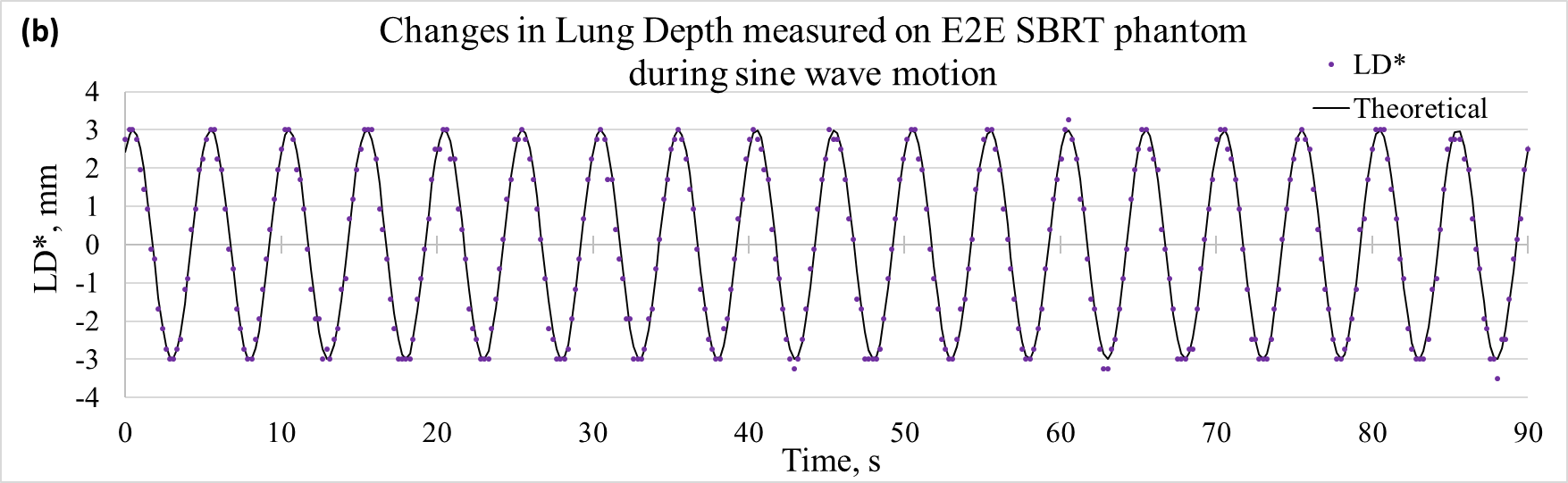


Fig. S2. (a) Changes in the skin distance (SD*) measured in images of the E2E SBRT phantom during the sine wave motion (time period 5 s, peak-to-peak amplitude 6 mm). (b) Changes in the lung depth (LD*).


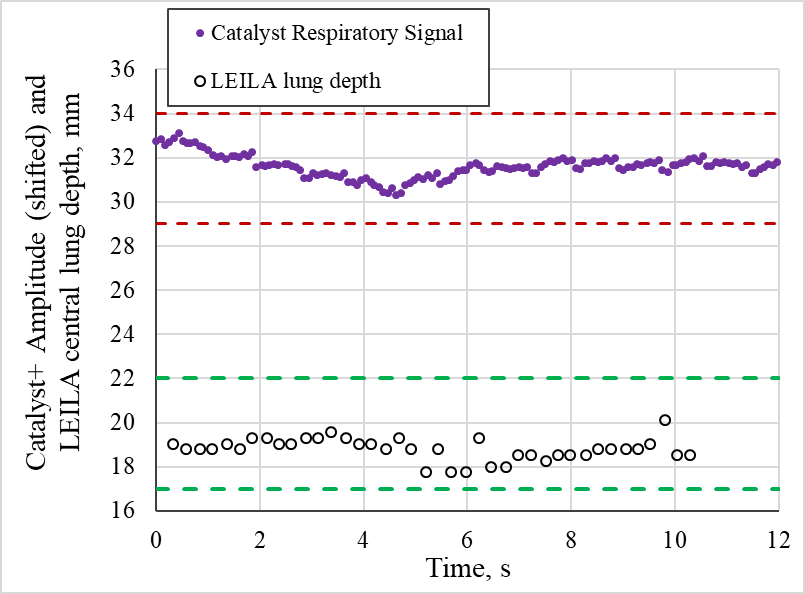


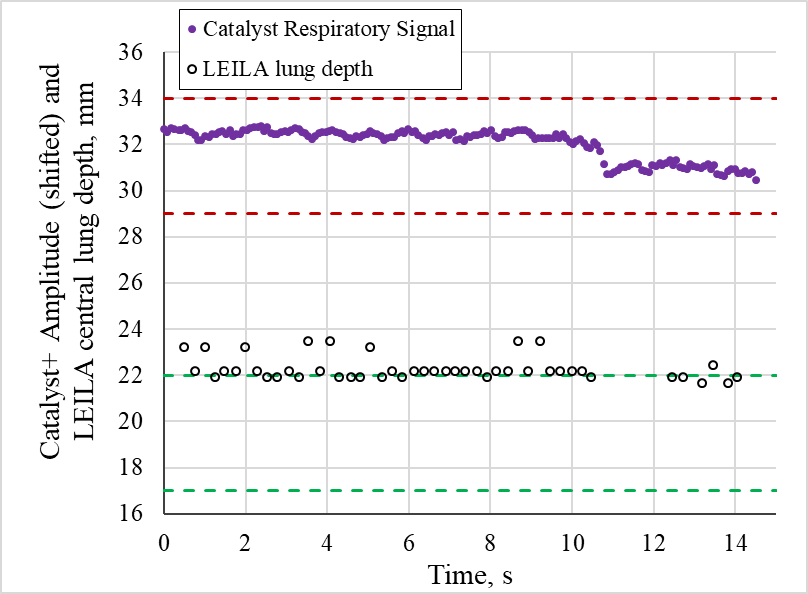


Fig. S3. The time traces of DIBH (deep inspiration breath-hold) signals recorded with Catalyst+ system and LEILA software (the central lung depth (CLD), open circles) during the beam-on time interval of beam 3 on different days for the same patient as in Fig. 10 of the main text. The 5 mm tolerance window for CLD for the treatment beams is from 17 to 22 mm. The tolerance window for CLD is shown by the green dashed lines. Initial values of the Catalyst+ amplitudes were shifted for convenience of comparison with CLD data.

*Section S4. The distance from the skin to the posterior field edge (the skin distance (SD) or the irradiated width) as a reliable breath hold indicator.*

The superior, central, and inferior lung depth (LD) and SD values were measured in 717 cine EPID images retrospectively using LEILA’s prototype software coded in MATLAB. The images were acquired in cine mode for an earlier study [14] and as such each represented averages of several EPID frames. These images were acquired during 15 fractions of DIBH tangential breast cancer RT of 12 different patients with 3 patients contributing 2 fractions each. Images with MLC leaves blocking the surface of the breast were excluded from the analysis and only images with medial-lateral (ML) views were examined to compare LDs and SDs obtained at one gantry angle.

The LD was measured as a distance between the posterior radiation field edge and the maximum pixel intensity of the lung–chest wall interface and the SD was measured as the distance from the breast surface to the posterior field edge (as shown in Figure 2 of the manuscript). The SD could be extracted from all images for all three locations; the LD was available for all images at the central line and at the superior line, but only for 83% of images at the inferior line. Using the variation of the quantity as a measure of reliability, the standard deviations (s.d., mm) of LD and SD were assessed for each fraction (Table S1).

Table S1. Standard deviation of LD and SD from medial-lateral (ML) images of one fraction.


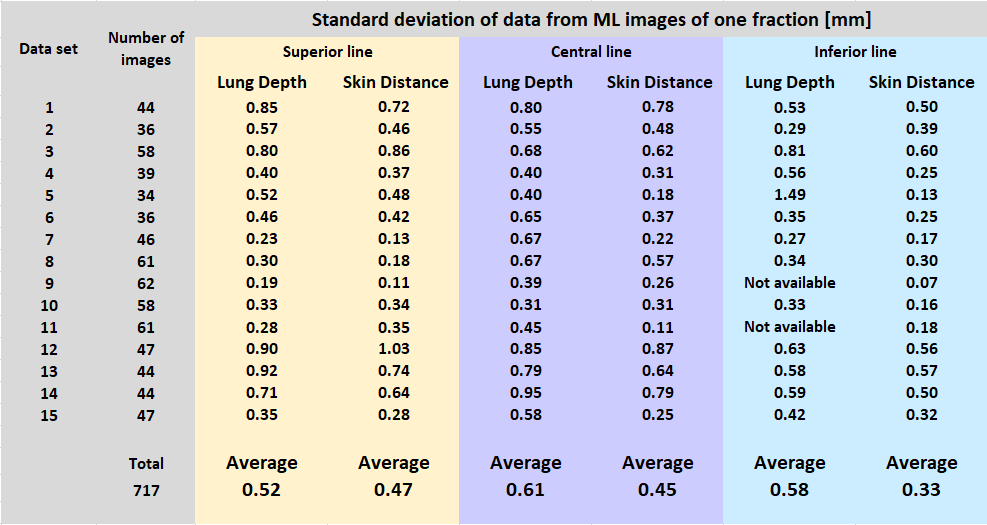


*At the superior line:* 11 of 15 data sets showed a higher s.d. for LD than for SD.

*At the central line:* 14 of 15 data sets showed a higher s.d. for LD than for SD.

*At the inferior line:* 14 of 15 data sets showed a higher s.d. for LD than for SD. LD was available for 83% of images.
